# Utilising previous radiographs: the principle of dose optimisation

**DOI:** 10.1259/bjrcr.20200018

**Published:** 2020-09-03

**Authors:** Farnaz Akbarian Tefaghi, Ashok Adams, Jimmy Makdissi

**Affiliations:** 1 Centre for Oral Bio-Engineering, Institute of Dentistry, Barts and the London School of Medicine and Dentistry Queen Mary University of London, London, United Kingdom; 2 The Royal London Hospital, London, United Kingdom; 3 Institute of Dentistry, Barts and the London School of Medicine and Dentistry, Queen Mary University of London, London, United Kingdom

## Abstract

**Objective:**

In dentistry, imaging is the most frequently used diagnostic tool. As a result, a steady increase in the use of imaging modalities are leading to an increase in healthcare cost and in patients’ radiation exposure.

**Results:**

67-year-old patient attended for a surgical removal of lower left third molar. A sectioned panoramic radiograph showed an incidental finding of a well-defined, unilocular radiolucency apical to the lower left second and third molars. This was partially superimposed over the outline of the ID canal. A Stafne’s bone cavity was considered as the most likely diagnosis. Further imaging was considered due to location not being fully below the ID canal as usually described in the literature. Reviewing previous imaging on PACS revealed the patient has had a CT angiogram of the head and neck 5 years prior. This showed a lingual bone defect of the surface of the mandible in the region of interest. The extension of the submandibular gland into the defect confirmed the likely nature of Stafne’s bone cavity.

**Conclusion:**

This case highlights the essential role of reviewing (if in the same practice) or requesting (from a different practice) previous images. The international Commission for Radiological Protection regularly publishes data relating to the principles of dose reduction; Justification, Optimisation and Limitation. All examinations have to be justified to ensure the benefit to the patient outweigh the risk and radiation should be kept as low as reasonably achievable.

## Summary

In dentistry, imaging is the most frequently used diagnostic tool. The easy access to medical and dental imaging has made us clinicians more accepting to requesting radiographic examinations. This has been coupled with recent advances in medical imaging technology including software reconstruction and detector resolution. As a result, a steady increase in the use of imaging modalities are leading to an increase in healthcare cost and patients’ radiation exposure.^1^

## Clinical presentation

We present a case where the use of previous imaging has aided the diagnosis and prevented unnecessary exposure to the patient.

**Figure 1.**
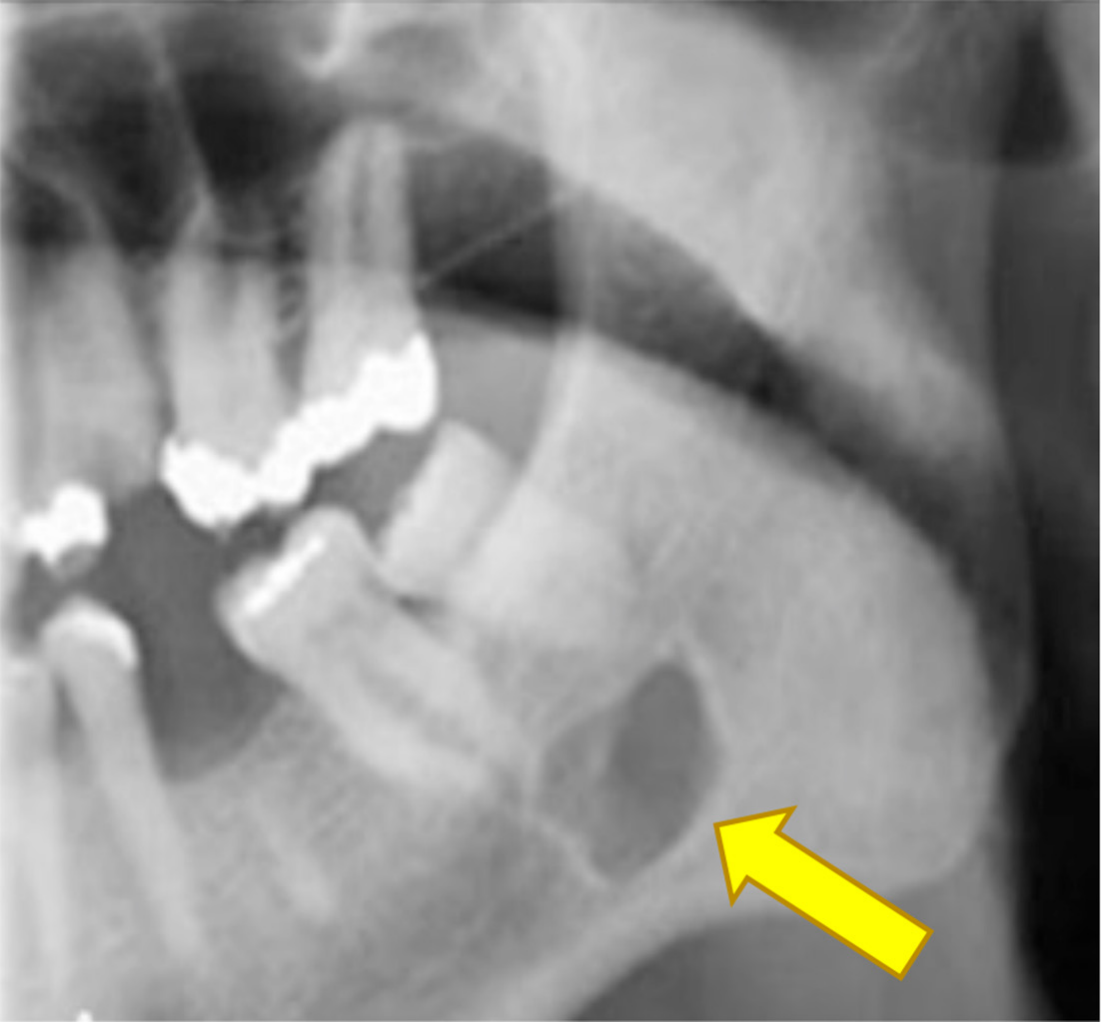
Sectioned panoramic radiograph.

A 67-year-old patient attended for a surgical removal of lower left third molar. A sectioned panoramic radiograph (Figure 1) showed an incidental finding of a well-defined, unilocular radiolucency measuring 10 × 7 mm apical to the lower left second and third molars. This was partially superimposed over the outline of the ID canal.

## Differential diagnosis

A Stafne’s bone cavity (SBC) was considered the most likely diagnosis.^2^ For further investigation, a 6 × 6 cm FOV (field of view) CBCT (cone beam computed tomography) was considered due to location not being fully below the ID canal as usually described in the literature.

### Investigations/imaging findings

**Figure 2.**
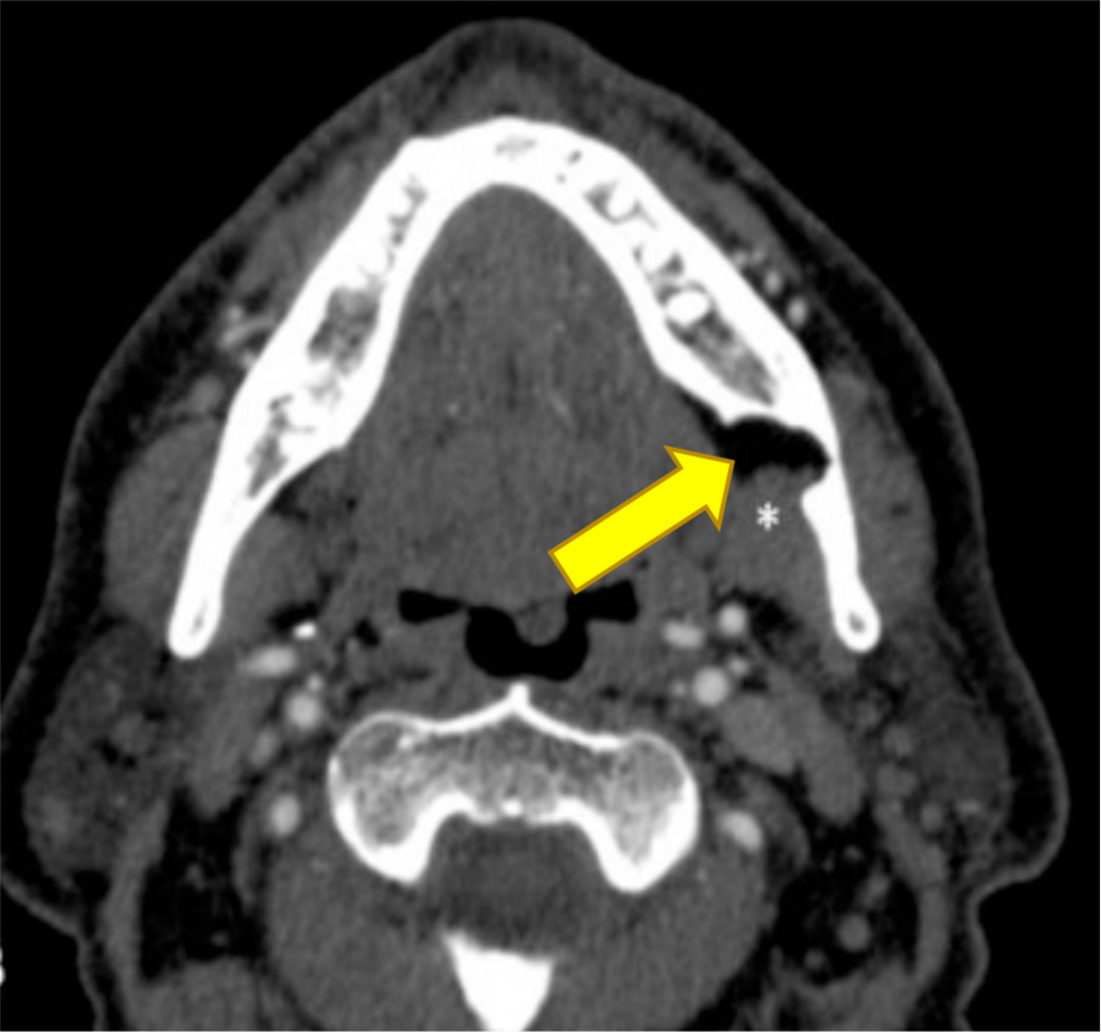
­Constructed Image of CT-Angiogram: The yellow arrow shows the extension of the submandibular gland into the defect

A radiologist opinion was sought. Reviewing previous imaging on PACS (Picture Archive and Communication System) revealed the patient has had a CT angiogram of the head and neck 5 years prior (Figure 2). As a result, no further imaging was needed and therefore the patient was saved from extra radiation of about 15–20 microsievert. This showed a lingual bone defect of the surface of the mandible in the region of interest. The extension of the submandibular gland into the defect confirmed the likely nature of SBC.^3,4^

**Figure 3.**
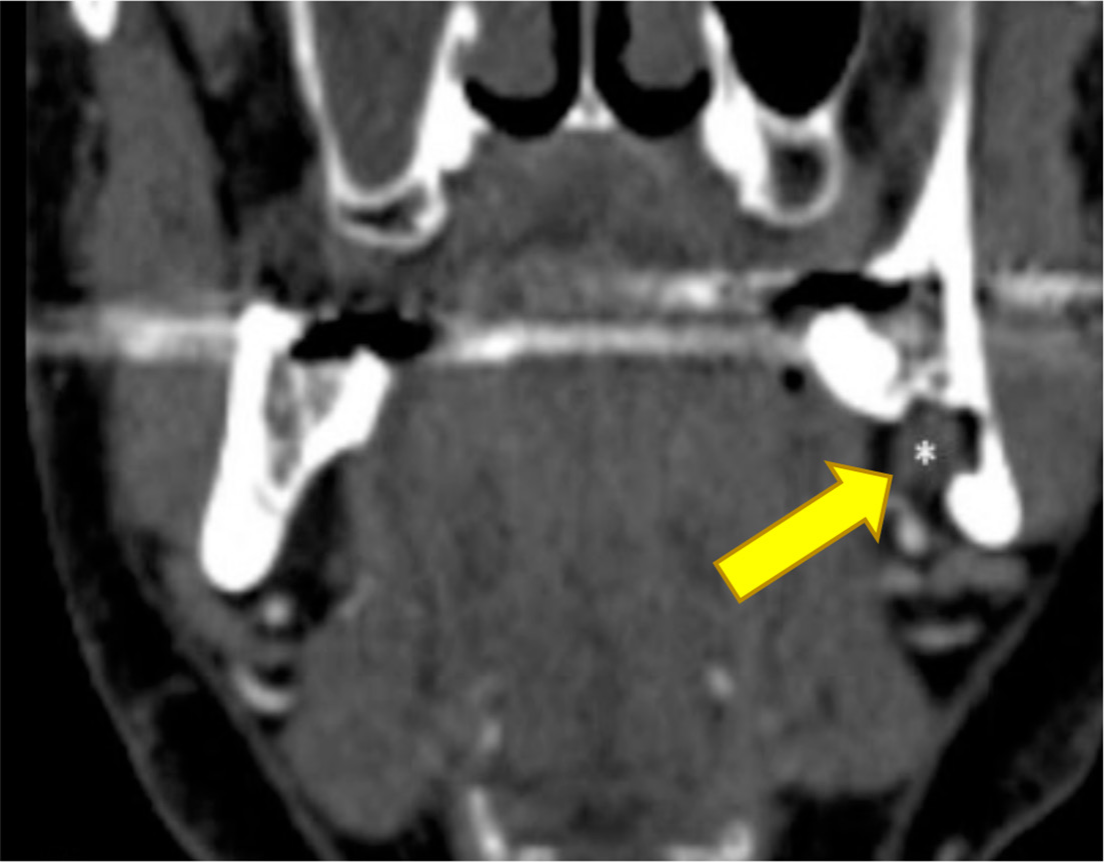
Constructed images of CT angiogram: The yellow arrow indicates the extension of the submandibular gland into the defect

**Figure 4.**
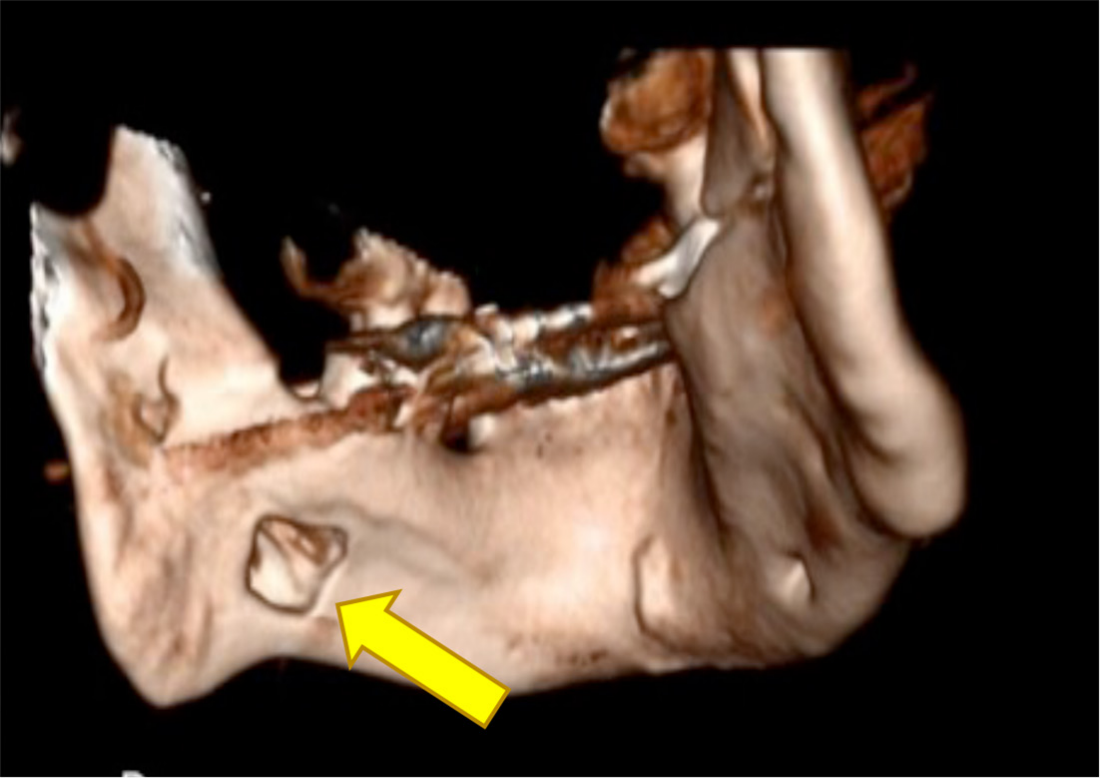
3D image of mandible (constructed from CT angiogram) yellow arrows show the position of the lesion. 3D, three-dimensional; SBC, Stafne’s bone cavity.

## Outcome and discussion

SBC is a benign condition also known as lingual mandibular bone defect (LMBD), idiopathic bone cavity, static bone defect or ectopic salivary gland. It describes a cavity on the lingual surface of the mandible often filled with normal salivary gland tissue, but occasionally they contain skeletal muscle, fibrous connective tissue, adipose tissue, lymphatic tissue and blood vessels.^5,6^

## Treatment

No further investigation and treatment were required.

## Learning points

Previous imaging has rendered the need for any new imaging unnecessary. This case highlights

- The essential role of reviewing (if in the same practice) or requesting (from a different practice) previous images.
- PACS is widely used in any Hospital in developed countries and therefore previously performed imaging procedures are easily accessible.
- The International Commission for Radiological Protection regularly publishes data relating to the principles of dose reduction; Justification, Optimization and Limitation.
- All examinations have to be justified to ensure the benefit to the patient outweigh the risk and should be optimised to secure radiation exposures are kept as low as reasonably achievable.^7^
